# Effects of Polyethylene Glycol Spacer Length and Ligand Density on Folate Receptor Targeting of Liposomal Doxorubicin In Vitro

**DOI:** 10.1155/2011/160967

**Published:** 2010-12-15

**Authors:** Kumi Kawano, Yoshie Maitani

**Affiliations:** Institute of Medicinal Chemistry, Hoshi University, Ebara 2-4-41, Shinagawa-ku, Tokyo 142-8501, Japan

## Abstract

The folate receptor is an attractive target for selective tumor delivery of liposomal doxorubicin (DXR) because it is abundantly expressed in a large percentage of tumors. This study examined the effect of polyethylene glycol (PEG) spacer length and folate ligand density on the targeting ability of folate-modified liposomes. Liposomes were modified with folate-derivatized PEG-distearoylphosphatidylethanolamine with PEG molecular weights of 2000, 3400, or 5000. The association of DXR-loaded liposomes with KB cells, which overexpress the folate receptor, was evaluated by flow cytometry at various ratios of folate modification. A low ratio of folate modification with a sufficiently long PEG chain showed the highest folate receptor-mediated association with the cells, but did not show the highest in vitro cytotoxicity. DXR release from folate-modified liposomes in endosomes might be different. These findings will be useful for designing folate receptor-targeting carriers.

## 1. Introduction

Antitumor drug delivery systems with nanoscopic dimensions have received much attention due to their unique accumulation behavior at the tumor site. Various nanoparticulate carriers such as liposomes, polymer conjugates, polymeric micelles, and nanoparticles are utilized for selective delivery of various anticancer drugs to tumors in a passive targeting manner [1]. However, a more effective and active targeting system is needed to enhance the uptake of drugs using nanocarriers into cancerous cells at the tumor site. 

Receptor-mediated endocytosis pathways have been exploited for tumor-specific targeting of nanocarriers and intracellular delivery of their contents. Modification of carriers with a ligand directed to an overexpressed receptor in cancer cells can improve selectivity and facilitate the movement of carriers into the intracellular compartment. One such candidate ligand is folic acid because the folate receptor-*α* is overexpressed in a number of human tumors, including ovarian, lung, brain, head and neck, and breast tumors [2–4]. Folic acid has been widely employed as a targeting moiety for various anticancer drugs through covalent conjugation to anticancer drugs and nanocarriers [5–8]. Liposomes modified with folic acid showed selective targeting toward human carcinomas along with enhancement of doxorubicin (DXR) cytotoxicity in vitro [9]. 

Ligand density per drug carrier and spacer length are important in designing suitable carriers for targeting. However, the optimal ligand density on liposomes is controversial. Different densities of folate in liposomes (ligand/total lipid molar ratio) ranging between 0.01% and 1.0% have been reported in the literature as sufficient to promote liposome binding to the folate receptor on cells [10–12]. These differences may be related to the accessibility of the folate ligand [13] or to the differences in the polyethylene glycol (PEG)-folate chemical linkage [14]. Because PEGylated liposomes, called sterically stabilized liposomes, reduce the association of liposome-modified ligands with their receptors by steric hindrance of the PEG polymer [13], we used non-PEGylated liposomes to examine the optimum number and spacer length of the targeting ligand.

In this study, folate-mediated association of DXR-loaded liposomes with human oral carcinoma KB cells, which overexpress the folate receptor, was evaluated in terms of PEG spacer length and the ratio of modification with the folate ligand. Enhanced association of DXR in KB cells was shown with an extremely low ratio of folate modification and a sufficiently long PEG spacer length, but high cytotoxicity of DXR was observed with a high ratio of folate modification.

## 2. Materials and Methods

### 2.1. Materials

Hydrogenated soybean phosphatidylcholine (HSPC), aminopoly(ethyleneglycol)-distearoylphosphatidylethanolamine (amino-PEG-DSPE, PEG mean molecular weight of 2000, 3400, and 5000), and methoxy-PEG_5000_-DSPE (mPEG_5000_-DSPE) were obtained from NOF Corporation (Tokyo, Japan). Cholesterol (Ch), doxorubicin (DXR) hydrochloride, folic acid, and HPLC-grade acetonitrile were purchased from Wako Pure Chemical Industries, Ltd. (Osaka, Japan). Folate-derivatized PEG-DSPE (F-PEG_2000_-, F-PEG_3400_-, and F-PEG_5000_-DSPE), which are conjugates of folic acid and amino-PEG-DSPE, were synthesized as reported previously [13, 15]. Ionophore A23187 and 1,1′-dioctadecyl-3,3,3′,3′-tetramethylindocarbocyanine perchlorate (DiI) were purchased from Sigma (St. Louis, MO, USA) and Lambda Probes and Diagnostics (Graz, Austria), respectively. Folate-deficient RPMI 1640 medium and fetal bovine serum (FBS) were obtained from Invitrogen Corp., (Carlsbad, CA, USA). Other reagents used in this study were reagent grade.

### 2.2. Preparation of Folate-Modified Liposomes

Liposomes were prepared from HSPC/Ch (55/45 mol/mol). All lipids were dissolved in chloroform, which was removed by evaporation. Lipophilic fluorescent marker DiI-labeled liposomes were prepared by the same procedure, but with the addition of DiI (0.4 mol% of total lipid) to the lipid mixture and without DXR loading. The film was hydrated with MgSO_4_ aqueous solution (300 mM, adjusted to pH 3.5 with HCl) and sonication. The resulting mean diameter of liposomes was about 130 nm, as determined by the dynamic light scattering method (ELS-800; Otsuka Electronics Co., Ltd., Osaka, Japan) at 25°C after diluting the liposome suspension with water. 

DXR was encapsulated into liposomes using the ionophore-mediated loading method [16, 17]. The MgSO_4_ gradient was formed by exchange of the external solution with sucrose buffer (300 mM sucrose, 20 mM HEPES, and 15 mM EDTA; pH 7.4) by gel filtration chromatography. Subsequent addition of ionophore A23187 to the liposome dispersion results in the outward movement of 1 metal cation in exchange for 2 protons, thus establishing a transmembrane pH gradient. A23187 was used at a concentration of 0.1 *μ*g/*μ*mol lipid and liposomes were incubated with the ionophore at 60°C for 5 min prior to the addition of drug. DXR was then added to the liposomes at a final drug-to-lipid ratio of 0.2 : 1 (wt/wt) and incubated at 60°C for 20 min. 

For comparison of loading procedures, DXR was encapsulated in liposomes by the pH gradient method [18]. Briefly, the lipid film was hydrated with citrate buffer (300 mM; pH 4.0) and sonicated. After the external pH was adjusted to 7.4, liposomes were incubated with DXR (drug : lipid = 0.2 : 1, wt/wt) at 60°C for 20 min. 

The folate ligand was inserted into preformed liposomes by the postinsertion technique [19]. Briefly, liposomes (DXR-loaded or DiI-labeled) were incubated with an aqueous dispersion of F-PEG-DSPE (from 0.01 to 1 mol% of total lipid) at 60°C for 1 h. In the case of unmodified liposomes (NF-L), water was added instead of F-PEG-DSPE solution. Liposomes modified with F-PEG_2000_-, F-PEG_3400_-, F-PEG_5000_-, or mPEG_5000_-DSPE will henceforth be designated as F2-L, F3-L, F5-L, and M5-L, respectively. After heating, the liposomes were cooled to room temperature. The suspension was then passed through a Sephadex G-50 column to remove any leaked DXR and unincorporated folate ligand. DXR loading efficiency was determined and significant DXR leakage was not observed with incubation of F-PEG-DSPE at these concentrations. DXR concentration was determined by measuring absorbance at 480 nm (UV-1700 Phamaspec, Shimadzu Corp., Kyoto, Japan).

### 2.3. In Vitro Assay for Drug Retention

The release of drug from the liposomes in phosphate-buffered saline (PBS, pH 7.4 or 5.0) was monitored by a dialysis method. The dialysis was done at 37°C using seamless cellulose tube membranes (Spectrum, Houston, TX, USA) with a molecular weight cutoff of 300,000 Da and PBS as the sink solution. The initial concentration of DXR-loaded liposomes was 0.2 mg/mL. The sample volume in the dialysis bag was 1 mL, and the sink volume was 100 mL. The concentration of drug was analyzed at various times points during dialysis.

### 2.4. Cellular Association of Liposomes Determined by Flow Cytometry

KB cells were obtained from the Cell Resource Center for Biomedical Research, Tohoku University (Miyagi, Japan). The cells were cultured in folate-deficient RPMI 1640 medium with 10% heat-inactivated FBS and kanamycin sulfate (50 *μ*g/mL) in a humidified atmosphere containing 5% CO_2_ at 37°C.

The cells were prepared by plating 3 × 10^5^ cells/well in a 12-well culture plate 1 day before the assay. The cells were incubated with DXR-loaded liposomes or DiI-labeled liposomes containing 20 *μ*g/mL DXR or 100 *μ*g/mL lipid diluted in 1 mL of serum-free medium for 2 h or the indicated time at 37°C. For free ligand competition studies, 1 mM folic acid was added to the medium. After incubation, the cells were washed with cold PBS (pH 7.4), detached with 0.02% EDTA-PBS, and then suspended in PBS containing 0.1% bovine serum albumin and 1 mM EDTA. The suspended cells were directly introduced into a FACSCalibur flow cytometer (Becton Dickinson, San Jose, CA) equipped with a 488 nm argon ion laser. Data for 10,000 fluorescent events were obtained by recording forward scatter, side scatter, and 585/42 nm fluorescence. The autofluorescence of cells incubated with serum-free medium without drug for 2 h was used as the control.

### 2.5. Cytotoxicity of Liposomes in KB Cells

KB cells were incubated with DXR-loaded liposomes (20 *μ*g/mL) for 2 h. After incubation, the cells were washed with cold PBS and cultured in fresh medium for 48 h. Cytotoxicity was determined using the WST-8 assay (Dojindo Laboratories, Kumamoto, Japan) based on enzymatic reduction of a tetrazolium salt, WST-8, to water-soluble formazan. The number of viable cells was then determined by absorbance at 450 nm.

## 3. Results and Discussion

### 3.1. Characterization of DXR-Loaded and Folate-Modified Liposomes

**Figure 1:**
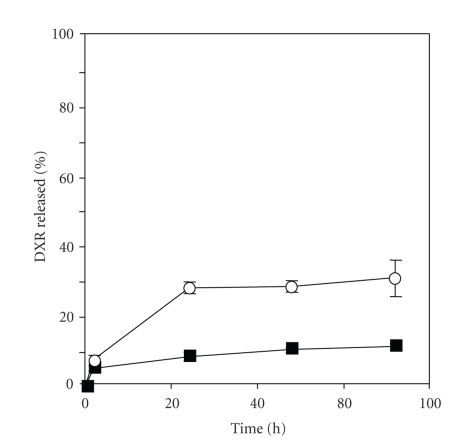
DXR release profile of liposomes loaded by the MgSO_4_/ionophore method (■) and pH gradient method using citrate buffer (○) in PBS (pH 7.4) at 37°C. Each value represents the mean ± SD (*n* = 3).

For efficient drug delivery to the target site, drugs should be stably entrapped in liposomes. In this study, an ionophore-mediated pH gradient method utilizing MgSO_4_ was applied to load DXR into liposomes because this method can effectively encapsulate drugs [17]. More than 95% of DXR was incorporated in liposomes using this system at a drug-to-total lipid ratio of 1 : 5 (wt : wt). The drug retention in the liposomes was examined by incubation in PBS (pH 7.4) at 37°C. For comparison, DXR-liposomes loaded by the remote loading method using citrate buffer were used. As shown in Figure 1, DXR-liposomes loaded using MgSO_4_ showed significantly lower DXR leakage during the 72-h incubation compared with those loaded using citrate buffer, indicating that the DXR-liposomes produced by the ionophore/MgSO_4_ loading method were more stable than those produced by the pH gradient method. Therefore, we applied the ionophore/MgSO_4_ method to load DXR into liposomes for evaluation of folate receptor-targeted liposomes. 

The average particle size of liposomes used in this study was approximately 130 nm, and the folate modification did not change the sizes of liposomes. More than 80% of folate ligand was inserted in liposomes at each ratio, which was confirmed after the separation of folate-modified liposomes by ultracentrifugation (100,000 × g, 1 h, 4°C).

### 3.2. Effects of Spacer Length and Modification Ratio of F-PEG-DSPE on Liposome Association with KB Cells

**Figure 2:**
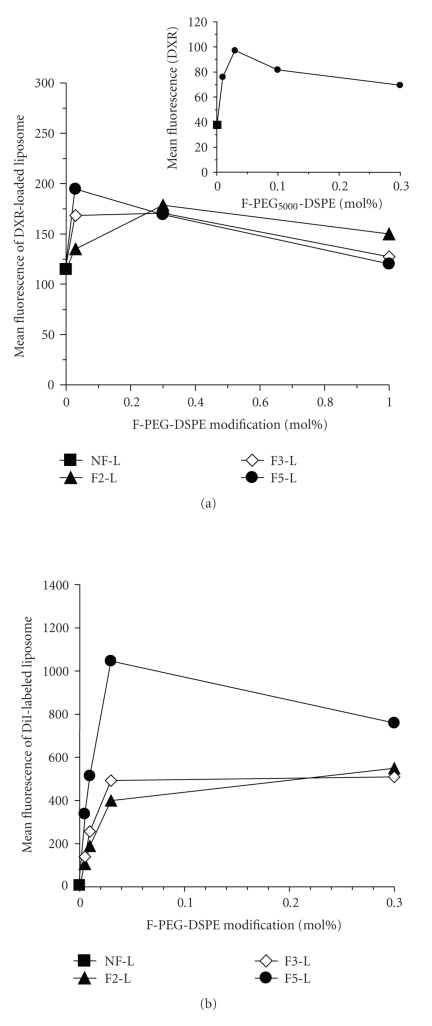
Association of folate-modified liposomes with KB cells with 2-h incubation was determined by fluorescence of DXR-loaded liposomes (a) and DiI-labeled liposomes (b) using flow cytometry. (a) Folate modification from 0.03 to 1.0 mol% of F2-, F3-, and F5-PEG-DSPE. Inset: F5-PEG-DSPE modification from 0.01 to 0.3 mol%. (b) Folate modification from 0.01 to 0.3 mol% of F2-, F3-, and F5-PEG-DSPE.

In this study, the cellular association of folate-modified liposomes was evaluated in KB cells with respect to PEG spacer length and modification ratio by flow cytometry based on DXR fluorescence (Figure 2(a)) and DiI-labeled liposomes (Figure 2(b)). Folate modification with F-PEG_2000_-, F-PEG_3400_-, or F-PEG_5000_-DSPE (F2-, F3-, and F5-L) at 0.03 to 1.0 mol% enhanced the cellular association compared to that of unmodified liposomes (NF-L), indicating that differences in the density and PEG spacer length of folate ligands resulted in different liposome associations with KB cells. The highest association of liposomes was observed with 0.03 mol% folate modification with the PEG_5000_ spacer, which was 1.7-fold and 160-fold higher than that of unmodified liposomes by measurement of DXR and DiI, respectively (Figure 2(a) inset and Figure 2(b)). The large discrepancy in the value might be due to the difference of distribution of DXR and DiI in liposomes, that is, DXR was entrapped in the water phase of liposomes, but DiI was incorporated in the liposomal membrane. Both the drug incorporated into liposomes and the lipid membrane of liposomes revealed a similar enhancement in association, suggesting that the DXR was associated in the liposomal form, not as drug released from liposomes.

**Figure 3:**
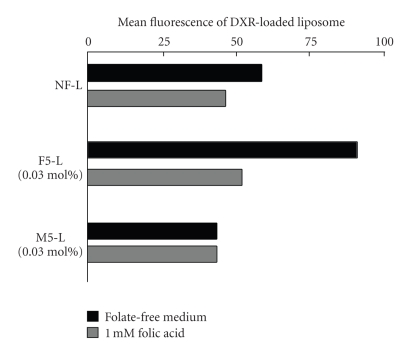
Association of DXR-loaded liposomes with KB cells with 1-h incubation was determined by flow cytometry. Cells were incubated with each liposome in folate-free medium or medium containing 1 mM folic acid.

Next, we confirmed whether folate might mediate cellular association with KB cells by mPEG-DSPE modification and free ligand competition (Figure 3). F5-L with 0.03 mol% folate modification showed higher cellular association than NF-L and M5-L (0.03 mol% mPEG-DSPE modification) did. Furthermore, the cellular association of F5-L could be blocked by 1 mM free folic acid and reduced to the level of NF-L. These results indicated that enhancement was due to folate-mediated cellular association. 

**Figure 4:**
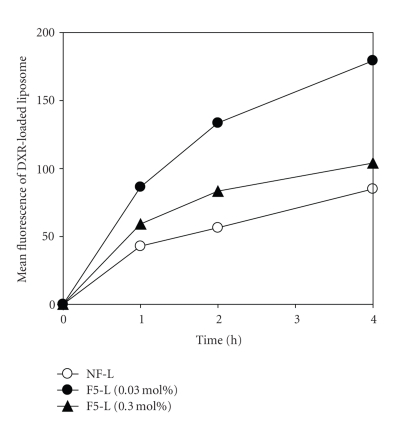
Cellular association of DXR-loaded liposomes with time was determined by flow cytometry. KB cells were incubated with F5-L modified at 0.03 or 0.3 mol% or without folate modification (NF-L).

The effect of incubation time on the cellular association of liposomes was then examined (Figure 4). As the incubation time increased, cellular associations increased and higher association was observed with F5-L modified at 0.03 mol% than at 0.3 mol%. Cellular association of liposomes modified at 0.3 mol% seemed to be saturated after a 2-h incubation. It has been reported that the folate receptor recycling system is downregulated as a result of satisfaction of the cellular folate requirement [11]. Therefore, liposomes modified with more folate ligands would lead to a larger intracellular folate content than those with fewer targeting ligands. Our data showed that liposomes containing fewer folate ligands per liposome had higher association efficiencies compared to liposomes containing large numbers of folate ligands per liposome. As a result, liposomes with minimal folate ligands may be efficient in enhancing drug accumulation in cells.

### 3.3. Effect of Folate Modification Ratio on Cytotoxicity

**Figure 5:**
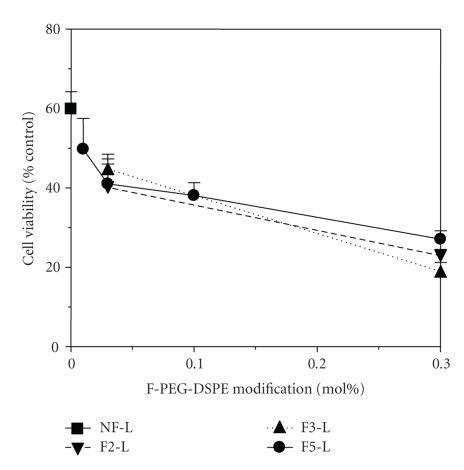
Cytotoxicity of DXR-loaded liposomes against KB cells. Cells were incubated with each liposome at a DXR concentration of 20 *μ*g/mL for 2 h, then in fresh medium for 48 h. Each value represents the mean ± SD (*n* = 3).

The effect of the folate modification ratio on cytotoxicity in KB cells was evaluated using the WST-8 assay (Figure 5). Cell viability was compared with untreated control. All folate-modified liposomes showed higher cytotoxicity than NF-L. The cytotoxicity of F5-L was sharply enhanced from 0 mol% to 0.03 mol% folate modification, which correlated with the result of their cellular associations (Figure 2). However, modification with concentrations greater than 0.03 mol% gently enhanced the cytotoxicity, which did not correlate with cellular associations. As in the case of F5-L, 0.3 mol% folate-modified F2-L and F3-L showed higher cytotoxicity than with 0.03 mol% modification.

**Figure 6:**
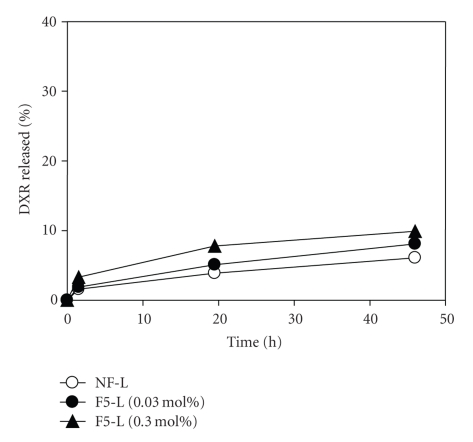
DXR release profile of folate-modified liposomes (F5-L) in PBS (pH 5.0) at 37°C. Each point represents the mean of 2 experiments.

The release of free drug from liposomes is involved in cytotoxicity or antitumor activity [20, 21]. Thus, we measured DXR release from liposomes with different modification ratios at pH 5.0, which resembled endosome content (Figure 6). Liposomes with high modification showed slightly higher drug release than those with low or no modification, although the release from all formulations was very low. Because DXR was stably loaded in the liposome using ionophore/MgSO_4_, it may be difficult to evaluate drug release differences under these conditions. Taken together, the enhanced cytotoxicity might reflect changes in drug release from liposomes by folate modification. Further evaluation of folate-modified drug carriers will be needed to optimize cellular association, cytotoxicity, and/or antitumor effects.

## 4. Conclusions

In this study, the effects of PEG spacer length and ligand density on folate receptor-targeted liposomes were evaluated. A low ratio of folate modification with a sufficiently long PEG chain (F-PEG_5000_-DSPE) increased folate receptor-mediated association, but a high ratio of folate modification enhanced in vitro cytotoxicity. This information will be useful for designing folate receptor-targeting carriers.
